# Ion Channel Activities in Neural Stem Cells of the Neuroepithelium

**DOI:** 10.1155/2012/247670

**Published:** 2012-07-03

**Authors:** Masayuki Yamashita

**Affiliations:** Department of Physiology 1, Nara Medical University, Shijo-cho 840, Kashihara 634-8521, Japan

## Abstract

During the embryonic development of the central nervous system, neuroepithelial cells act as neural stem cells. They undergo interkinetic nuclear movements along their apico-basal axis during the cell cycle. The neuroepithelial cell shows robust increases in the nucleoplasmic [Ca^2+^] in response to G protein-coupled receptor activation in S-phase, during which the nucleus is located in the basal region of the neuroepithelial cell. This response is caused by Ca^2+^ release from intracellular Ca^2+^ stores, which are comprised of the endoplasmic reticulum and the nuclear envelope. The Ca^2+^ release leads to the activation of Ca^2+^ entry from the extracellular space, which is called capacitative, or store-operated Ca^2+^ entry. These movements of Ca^2+^ are essential for DNA synthesis during S-phase. Spontaneous Ca^2+^ oscillations also occur synchronously across the cells. This synchronization is mediated by voltage fluctuations in the membrane potential of the nuclear envelope due to Ca^2+^ release and the counter movement of K^+^ ions; the voltage fluctuation induces alternating current (AC), which is transmitted via capacitative electrical coupling to the neighboring cells. The membrane potential across the plasma membrane is stabilized through gap junction coupling by lowering the input resistance. Thus, stored Ca^2+^ ions are a key player in the maintenance of the cellular activity of neuroepithelial cells.

## 1. Introduction

During the embryonic development of the central nervous system, cells in the neuroepithelium act as neural stem cells. The neuroepithelium forms the neural tube, from which the central nervous system including the spinal cord, retina, and brain is derived. The neuroepithelial cell has a polarized structure: the apical process faces the ventricle, and the furthest portion of the basal process makes contact with the basement membrane. This contact is necessary for the cell to undergo interkinetic nuclear movement along the apico-basal axis during the cell cycle [1–4]. Neuroepithelial cells in S-phase synthesize DNA in their basal region, followed by the movement of the soma towards the apical region prior to cell division during M-phase [3, 4].

**Figure 1:**
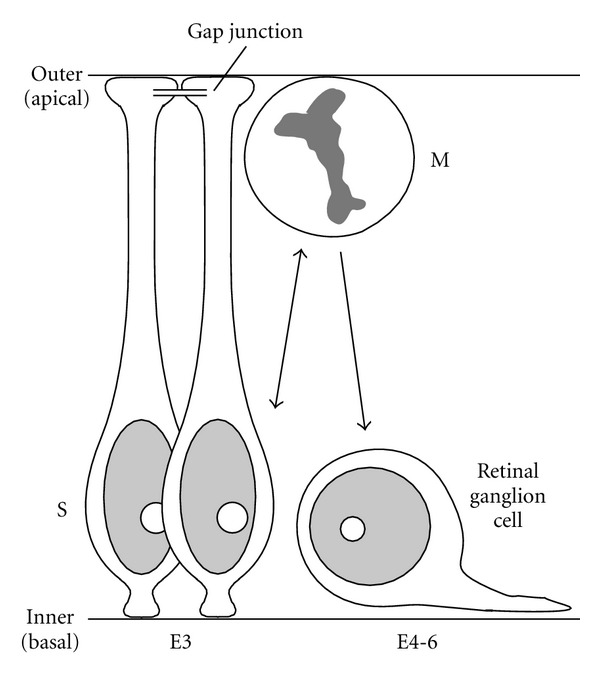
Schematic drawings of retinal neuroepithelial cells in cell cycle and the first differentiated neuron, a retinal ganglion cell. In chick embryo, the retina is composed almost homogeneously of neuroepithelial cells on embryonic day 3 (E3). The retinal ganglion cells are born mainly at E4–6. S: S-phase; M: M-phase. The outer (apical) surface faces the space that is continuous with the ventricle. This figure is cited from [5].

**Figure 2:**
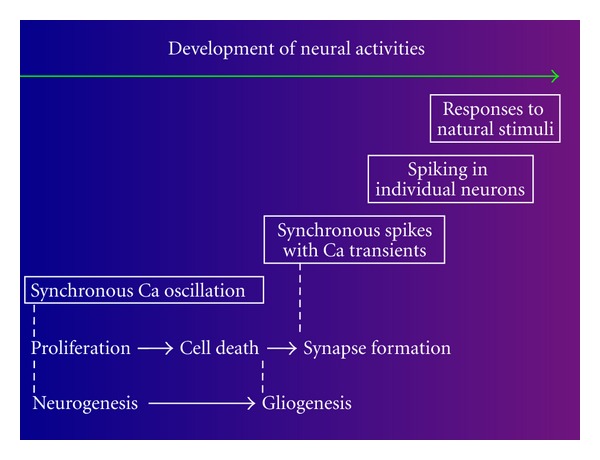
Developmental changes in neural activities. Self-renewing neuroepithelial cells show spontaneous, synchronous calcium oscillations. Newborn neurons show synchronous burst spike discharges before synapse formation. This figure is cited from [6].

To study ion channel activities in the neuroepithelial cell, the retinal neuroepithelium is a suitable model because the retina can be isolated from an optic cup at early stages of embryonic development. The neural tube evaginates laterally to form two optic vesicles, each of which invaginates to form an optic cup. The neuroepithelium on the inner wall of the optic cup becomes the retina.  Figure 1 shows the retinal neuroepithelial cells that are undergoing interkinetic nuclear movements and the first differentiated neuron, a retinal ganglion cell. Developmental changes in the cellular activity are summarized as a time diagram in Figure 2. Studies of the retinal neuroepithelial cells have revealed that various ion channels are assembled in these cells and are important for the maintenance of the cellular activity of neuroepithelial cells. The current paper is intended to survey and discuss the functional roles of ion channels found in the retinal neuroepithelial cell, as well as in other neural tube stem cells, and the cell line.

## 2. Channel-Mediated Release of Ca^2+^ from Intracellular Ca^2+^ Stores

Neuroepithelial cells show a robust response to the activation of G protein-coupled receptors (GPCRs) including muscarinic acetylcholine receptors [7], P2Y purinoceptors [8], and lysophosphatidic acid receptors [9], which leads to increases in intracellular Ca^2+^ concentrations ([Ca^2+^]). Activation of these GPCRs leads to the production of inositol 1,4,5-trisphosphate (InsP_3_) from phosphatidylinositol 4,5-bisphosphate (PIP_2_) via the phospholipase C enzyme. InsP_3_ activates the InsP_3_ receptor Ca^2+^ channel to cause the release of Ca^2+^ from intracellular Ca^2+^ stores (Ca^2+^ mobilization) [10]. Another type of Ca^2+^ releasing channel, namely ryanodine receptor channel, is unlikely to be functioning in the retinal neuroepithelial cell because no response was evoked by caffeine (an activator of ryanodine receptor) [8]. 

Using confocal fluorescence microscopy and a Ca^2+^-sensitive fluorescent indicator, it was shown that Ca^2+^ mobilization is dependent upon the cell cycle [11]. Increases in intracellular [Ca^2+^] occur in the nucleoplasm of S-phase cells, of which somata are localized to the basal region of the developing neuroepithelium. In contrast, Ca^2+^ mobilization decreases in M-phase cells, which are located in the apical region. In newborn retinal ganglion cells, which migrate to the basal region (Figure 1), Ca^2+^ mobilization is also reduced. These results suggest that the rise in nucleoplasmic [Ca^2+^] is necessary for DNA synthesis during S-phase and that the Ca^2+^ mobilization system is less active following neuronal differentiation [11]. It is not clear whether other phases in cell cycle (i.e., G_1_ or G_2_) show Ca^2+^ increases.

The GPCR-mediated Ca^2+^ response of the neuroepithelial cell also depends upon the developmental stage of the retina. On embryonic day 3 (E3), nearly all the cells in the neural retina arise from the self-renewal of the neuroepithelial cells (Figure 1), and the Ca^2+^ mobilization is most robust at this stage. Retinal ganglion cells and other types of neurons are born between E4 and E8, and then neurogenesis ceases [12]. Between E3 and E8, the Ca^2+^ response declines in parallel with the decreasing proliferative activity of the retinal cells [7–9, 13]. This developmental profile suggests that Ca^2+^ mobilization may be critical for the proliferation of neuroepithelial cells. In support of this concept, studies using retinal cell cultures have shown that the release of Ca^2+^ from intracellular Ca^2+^ store is essential for DNA synthesis in these cells [14].

Increases in Ca^2+^ mobilization also occur spontaneously as synchronized Ca^2+^ oscillations in the retinal neuroepithelial cells [15] (Figure 2). GPCRs, such as P2Y purinoceptors, may be constitutively activated by ambient ATP, which is released in both an autocrine and paracrine manner [16]. The activation of P2Y purinoceptors has also been shown to promote the proliferation of retinal neuroepithelial cells [16–18]. The inhibitory effects of P2 antagonists on the proliferation of retinal neuroepithelial cells suggest that endogenous ATP activates P2Y purinoceptors constitutively [16]. The mechanism underlying the synchronization of Ca^2+^ oscillations is discussed later in this text. 

Increases in the intracellular [Ca^2+^] of S-phase cells may be involved in the activation of Ca^2+^-dependent nuclear signaling for proliferation. It has been suggested in various cell types that calcineurin, a Ca^2+^/calmodulin-dependent phosphatase, dephosphorylates the transcription factor NFAT (nuclear factor of activated T-lymphocytes), which regulates cell cycle progression [19, 20].

## 3. Store-Operated Ca^2+^ Entry

The release of Ca^2+^ from intracellular Ca^2+^ stores has been shown to instantaneously induce an influx of extracellular Ca^2+^. This Ca^2+^ influx is called capacitative or store-operated Ca^2+^ entry, and is necessary for the replenishment of the intracellular Ca^2+^ stores [21, 22]. This type of Ca^2+^ influx also occurs in the retinal neuroepithelial cell [23]. 

The store-operated Ca^2+^ entry in the retinal neuroepithelial cell has also been shown to decline as the cell becomes increasingly differentiated [13, 23]. Store-operated Ca^2+^ entry has also been shown to be essential for DNA synthesis in cultured retinal cells [14]. In a culture model of neural stem cells, neuroblastoma × glioma NG108-15 cell line, store-operated Ca^2+^ entry is most frequent in proliferation, as opposed to neuronal differentiation, medium [24]. It has been suggested that the store-operated Ca^2+^ entry is also involved in the Ca^2+^-regulated transcription pathways for cell cycle progression via the activation of calcineurin and NFAT [19, 20].

The channels responsible for capacitative Ca^2+^ entry have been supposed to be transient receptor potential (TRP) channels [21, 22]. It is now evident that the STIM molecules function as Ca^2+^ sensors within the endoplasmic reticulum and the orai proteins function as the channel for Ca^2+^ influx [21, 22]. The specific channels involved in the store-operated Ca^2+^ entry in the neuroepithelial cell have not yet been identified.

## 4. Ligand-Gated Channels

Retinal neuroepithelial cells also express ligand-gated channels. Application of the neurotransmitter gamma-aminobutyric acid (GABA) causes a strong depolarization that leads to the activation of L-type Ca^2+^ channels, allowing Ca^2+^ influx [25]. The GABA-induced depolarization is due to the efflux of Cl^−^ ions through GABA_A_ receptor channels, since the intracellular [Cl^−^] in the retinal neuroepithelial cell is higher than that in a mature neuron and the equilibrium potential of Cl^−^ is more positive than the resting membrane potential [25].

The GABA-induced depolarization and Ca^2+^ influx through voltage-dependent Ca^2+^ channels have been shown to inhibit the DNA synthesis in cortical progenitor cells [26] and the cell cycle progression in neuronal precursors from striatum [27]. On the contrary, by causing influx of Cl^−^ ions and hyperpolarization, GABA has been shown to negatively control the proliferation in embryonic stem (ES) cells and neural crest stem (NCS) cells [28].

In addition to GABA, ATP depolarizes retinal neuroepithelial cells by activating P2X purinoceptor channels as revealed by intracellular recording from these cells (unpublished observation).

## 5. Gap Junctions as Stabilizers of Membrane Potential

Neuroepithelial cells adhere to each other through gap junctions, which are located at the apical process of the cell [29]. This gap junction coupling between retinal neuroepithelial cells was demonstrated by the intracellular injection of a fluorescent dye and the subsequent dye diffusion (dye coupling) [5]. Following application of the gap junction channel blocker carbenoxolone during intracellular recording from retinal neuroepithelial cells, it was demonstrated that the input resistance was dramatically increased. In addition, the membrane potential was depolarized and rendered unstable showing fluctuations during the recording in the presence of the blocker [5]. These results suggest that the gap junction coupling stabilizes the resting membrane potential of the neuroepithelial cell by lowering the input resistance. This characteristic of gap junction coupling in the neuroepithelial cell may underlie the maintenance of the driving force for Ca^2+^ influx during store-operated Ca^2+^ entry and the prevention of the excess, continuous influx of Ca^2+^ through L-type Ca^2+^ channels.

## 6. Na^+^, Ca^2+^, and K^+^ Channels in the Plasma Membrane

The neuroepithelial cell shows epithelial features including not only the polarized structure but also physiological properties. Studies of the chick retinal neuroepithelium and the amphibian neural tube have shown that neuroepithelial cells are nonexcitable [5, 30]. Instead of voltage-dependent Na^+^ channels, amiloride-sensitive epithelial-type Na^+^ channels are present in cells of the neural tube [31]. These channels allow a continuous influx of Na^+^ from the ventricular space, and Na^+^-K^+^ pumps extrude Na^+^ ions from the cell to generate the transneural tube potential (a lumen-negative DC potential) in the amphibian embryo [31]. An extracellular DC potential was also observed within the retinal neuroepithelium (unpublished observation). The polarized transport of Na^+^ from the ventricular space may contribute to the establishment of the DC potential. The retinal neuroepithelium is an electrically tight epithelium since the extracellular resistance is extremely high (≥300 M*Ω*) in the middle region of the retinal neuroepithelium (unpublished observation).

Voltage-gated Ca^2+^ channels are also present in the retinal neuroepithelial cell. Ca^2+^-sensitive fluorescence measurements revealed the presence of L-type channels [25]. The L-type Ca^2+^ channel allows a continuous Ca^2+^ influx when the cell is depolarized with a high concentration of extracellular K^+^ [25]. Since retinal neuroepithelial cells are interconnected through gap junctions, lowering the input resistance [5], it seems likely that these channels are activated only when a mass of cells are depolarized. A single neuroepithelial cell does not generate any spike-like potential, even following the injections of a strong depolarizing current [5]. Thus, the role of the L-type Ca^2+^ channel in these cells remains to be clarified. It can be supposed that if L-type Ca^2+^ channels are activated by GABA-induced depolarization after losing gap junction, the Ca^2+^ influx through these channels may inhibit cell cycle progression, as revealed in neural progenitor cells [26, 27]. It has also been shown that the influx of Ca^2+^ through L-type Ca^2+^ channels prevents apoptosis in culture models of neuronal death [32]. 

BK (big potassium, Ca^2+^- and voltage-dependent potassium) channels in the plasma membrane are activated by increases in intracellular [Ca^2+^], which may be caused by Ca^2+^ influx through voltage-dependent Ca^2+^ channels during depolarization. Cells expressing BK channels in the plasma membrane show a voltage sag in response to a depolarizing current injection. This is the case in newborn retinal ganglion cells, which do not yet generate a tetrodotoxin-sensitive Na^+^-dependent action potential [5]. The newborn ganglion cell loses its gap junction coupling, thus the input resistance is increased and the voltage response in the cell is rendered visible [5]. The voltage sag in response to a depolarizing current injection is also observed in retinal neuroepithelial cells in which the gap junction channels are blocked by carbenoxolone [5]. These data suggest that BK channels are also present in the plasma membrane of retinal neuroepithelial cells. It seems likely that the BK channels in the plasma membrane repolarize the membrane potential when intracellular [Ca^2+^] is increased.

## 7. BK Channels in the Nuclear Envelope and Endoplasmic Reticulum

Intracellular Ca^2+^ stores are comprised of the endoplasmic reticulum and the nuclear envelope [33, 34]. The lumen of the endoplasmic reticulum is continuous with the space between the outer and inner nuclear membranes [35]. Ca^2+^ ions are actively transported into this lumen by Ca^2+^ pumps. 

The release of Ca^2+^ from intracellular stores via InsP_3_ receptor channels leads to a charge movement across the store membrane from the lumen to the cytoplasm or to the nucleoplasm, which are electrically interconnected through low-resistance nuclear pores [36]. This charge movement should lead to a negative shift in the luminal potential [37]. Simultaneous measurements of the nuclear membrane potential and intracellular [Ca^2+^] revealed that the potential of the nuclear membrane changes in a biphasic manner together with the activation of GPCRs, leading to a transient hyperpolarization and a sustained depolarization associated with the release of Ca^2+^ and the electrogenic pumping of Ca^2+^ ions, respectively [38].

To maintain the driving force for Ca^2+^ release from intracellular stores, the counter movement of ions, such as K^+^, across the store membrane is absolutely necessary; without counter ion movement, the luminal potential hyperpolarizes to the equilibrium potential of Ca^2+^ and the driving force for Ca^2+^ release is lost [37]. In the sarcoplasmic reticulum, TRIC (trimeric intracellular cation) channels mediate the counter ion movement [39].

Patch clamp recordings from the nuclear envelope membrane have shown that this membrane contains BK channels and that these BK channels are activated by positive changes in the luminal potential (depolarization) and by an increase in the luminal [Ca^2+^] [15, 40]. Such voltage and Ca^2+^ dependence may suggest that hyperpolarization caused by the release of Ca^2+^ and a decrease in the luminal [Ca^2+^], together lead to the closure of the BK channel and cessation of Ca^2+^ release from the lumen. This has been proposed to be a hypothetical underlying mechanism to explain the “quantal” manner of Ca^2+^ release, in which Ca^2+^ release terminates after a rapid release of a fraction of stored Ca^2+^ [41].

## 8. Nuclear Envelope Potential and the Synchronization of Cellular Activity

Spontaneous Ca^2+^ oscillations occur synchronously across the cells in the retinal neuroepithelium, while agonist-induced [Ca^2+^] rises occur asynchronously [15]. The mechanism underlying the synchronization of Ca^2+^ oscillations has been a matter of debate [42]. The diffusion of InsP_3_ or Ca^2+^ itself through gap junction channels is unlikely to synchronize a [Ca^2+^] rise, because an agonist-induced Ca^2+^ release is not synchronized even with the application of a supramaximal concentration of the agonist [15, 42]. Instead, a capacitative, or AC (alternating current), electrical coupling model has been proposed [6]. In this model, the efflux of Ca^2+^ from intracellular stores and the counter influx of K^+^ into the store lumen cause alternating voltage changes across the store membrane, and this voltage fluctuation induces AC currents. In the neuroepithelial cell, the outer nuclear membrane is closely apposed to the plasma membrane, and the cells are tightly packed [6, 15, 38]. Thus, it is plausible that the voltage fluctuation in the nuclear envelope potential is synchronized across the cells by the AC current being transferred in series via the capacitance of the outer nuclear membrane and the plasma membrane [6, 41]. Real-time confocal fluorescence measurements using an organelle-specific voltage-sensitive dye revealed that the nuclear membrane potential generates spontaneous repeats of high frequency (100–300 Hz) bursts of fluctuations in potential [38]. Furthermore, oscillatory changes in nuclear membrane potential underlie spike burst generation in developing neurons prior to synapse formation [38]. These results support the capacitative coupling model, in which voltage fluctuations in the nuclear membrane potential synchronize Ca^2+^ release across the population of cells and also function as a current noise generator to cause synchronous burst spike discharges in the neurons at an early stage of neural development [6, 38].

## 9. Concluding Remarks

Table 1 summarizes the different types and functions of ion channels expressed by neuroepithelial cells. While neurons use voltage changes across the plasma membrane, such as action potentials and synaptic potentials, for intercellular communication, the neuroepithelial cell uses stored Ca^2+^ ions to enable proliferation as well as the synchronization of Ca^2+^ oscillations. This synchronization is mediated by voltage fluctuations in the membrane potential of the nuclear envelope that allow capacitative (AC) electrical coupling between the cells. The membrane potential across the plasma membrane is stabilized via gap junction coupling, which lowers the input resistance of the neuroepithelial cell. BK-type potassium channels may also contribute to the stabilization of the plasma membrane potential when intracellular [Ca^2+^] is increased. Thus, stored Ca^2+^ ions are a key player in the maintenance of the cellular activity of neuroepithelial cells.

## Figures and Tables

**Table 1:** Neuroepithelial-cell-ion channels and their functions.

Type of ion channel	Function of ion channel
Ion channels in the plasma membrane	
Gap junction channel	Stabilizing membrane potential by lowering input resistance
Store-operated Ca^2+^ entry channel (TRP or Orai)	Replenishing Ca^2+^ stores after Ca^2+^ release
Epithelial Na^+^ channel	Continuous Na^+^ influx from ventricular space to generate DC potential
L-type voltage-dependent Ca^2+^ channel	Ca^2+^ influx by depolarization
BK channel	Repolarizating after depolarization and intracellular [Ca^2+^] rise
GABA_A_ receptor channel	Depolarizing in response to GABA
P2X purinoceptor channel	Depolarizing in response to ATP

Ion channels in the nuclear envelope and the endoplasmic reticulum	
InsP_3_ receptor channel	Ca^2+^ release by activation of muscarinic acetylcholine receptor, P2Y purinoceptor, and lysophosphatidic acid receptor
BK channel	Counter ion movement for Ca^2+^ release to maintain driving force for Ca^2+^ release
